# Summer Rains and Dry Seasons in the Upper Blue Nile Basin: The Predictability of Half a Century of Past and Future Spatiotemporal Patterns

**DOI:** 10.1371/journal.pone.0068461

**Published:** 2013-07-15

**Authors:** Per-Erik Mellander, Solomon G. Gebrehiwot, Annemieke I. Gärdenäs, Woldeamlak Bewket, Kevin Bishop

**Affiliations:** 1 Agricultural Catchments Programme, Teagasc, Johnstown Castle Environmental Research Centre, Wexford, County Wexford, Ireland; 2 Department of Aquatic Sciences and Assessment, Swedish University of Agricultural Sciences, Uppsala, Sweden; 3 Department of Soil and Environment, Swedish University of Agricultural Sciences, Uppsala, Sweden; 4 Department of Geography and Environmental Studies, Addis Ababa University, Addis Ababa, Ethiopia; 5 Ethiopian Institute of Water Resources, Addis Ababa University, Addis Ababa, Ethiopia; 6 Department of Earth Sciences, Uppsala University, Uppsala, Sweden; Plymouth University, United Kingdom

## Abstract

During the last 100 years the Ethiopian upper Blue Nile Basin (BNB) has undergone major changes in land use, and is now potentially facing changes in climate. Rainfall over BNB supplies over two-thirds of the water to the Nile and supports a large local population living mainly on subsistence agriculture. Regional food security is sensitive to both the amount and timing of rain and is already an important political challenge that will be further complicated if scenarios of climate change are realized. In this study a simple spatial model of the timing and duration of summer rains (Kiremt) and dry season (Bega), and annual rain over the upper BNB was established from observed data between 1952 and 2004. The model was used to explore potential impacts of climate change on these rains, using a down-scaled ECHAM5/MP1-OM scenario between 2050 and 2100. Over the observed period the amount, onset and duration of Kiremt rains and rain-free Bega days have exhibited a consistent spatial pattern. The spatially averaged annual rainfall was 1490 mm of which 93% was Kiremt rain. The average Kiremt rain and number of rainy days was higher in the southwest (322 days) and decreased towards the north (136 days). Under the 2050–2100 scenario, the annual mean rainfall is predicted to increase by 6% and maintain the same spatial pattern as in the past. A larger change in annual rainfall is expected in the southwest (ca. +130 mm) with a gradually smaller change towards the north (ca. +70 mm). Results highlight the need to account for the characteristic spatiotemporal zonation when planning water management and climate adaptation within the upper BNB. The presented simple spatial resolved models of the presence of *Kiremt* and annual total rainfall could be used as a baseline for such long-term planning.

## Introduction

The Blue Nile Basin (BNB) supplies over two-thirds of the water to the Nile at Aswan in Egypt, and supports a large population living mainly on subsistence agriculture. Even if agriculture is the largest sector in Ethiopia, it is dominated by small scale farming with a low productivity [1]. The highlands of the upper BNB are vulnerable to the negative impact that a variable climate may have [2] as the effects of climate change on rainfall and temperature in the region will have major implications for both regional food security [3] as well as the transboundary water supply with all that entails for [4] Sudan and Egypt. The importance of resolving conflicting interests has led to many diplomatic initiatives over the last century, and one of the most recent is the Cooperative Framework Agreement to develop resources of the Nile for mutual benefit [5].

The limited availability of water resources during the dry season in Ethiopia has been exacerbated by the characteristic climate of the region with a long dry season each year [6] in conjunction with a population increase and human impacts on the landscape, such as erosion associated with land degradation. The boundary conditions for sustainability in the BNB are thus closely linked to the climate as the productivity of agriculture is sensitive to the timing as well as the amount of rainfall. While temperatures in Ethiopia have increased over the last decades [7], average annual rainfall has not changed over 40 years [8] but there have been inter-decadal variability [6], [7], [9].

The seasons in the region are commonly named with respect to their rainfall; *Bega* (in Amharic) means the dry winter season (October – February); *Belg* the small rains of spring (March – May); and *Kiremt* the wet summer season (June – September). By far the most important season is the *Kiremt,* accounting for about three quarters of total annual rainfall. Spatiotemporal patterns of rainfall over BNB are formed by the north-ward migration of the Inter Tropical Convergence Zone (ITCZ).

Decadal frequencies have been observed in the *Kiremt* rains due to anomalous north-south displacement of the ITCZ. This is believed to be associated to the interaction of the Walker and the Hadley cells over Africa [9]. The migration of the ITCZ is followed by subtropical high pressure systems developed (St. Helena, and Mascarene) and southwesterly winds carrying moisture from the Congo Basin that are subsequently released over the Ethiopian highlands [10]. The southwestern part of the BNB is open for westerly, low intensity advective rains for a longer time than the northeast. The quasi-permanent high pressure systems over the south Atlantic and south Indian Ocean together with the development of the tropical easterly jet and the East Africa Low Level Jet (also called Somali jet), further affect the quality of the *Kiremt* [11], [12], [13]. The daily high intensity rainfall of the *Kiremt* has a greater spatial variability than the low intensity events [14]. The overall annual spatial variability has a strong southwest to northeast component with longer duration of rains giving higher annual amounts in the southwest (*ca.* 2200 mm) and a shorter rain period giving lower amounts in the northeast (*ca.* 800 mm [15], [16]). The spatial pattern of the *Bega* may be seen as an “inverse” of *Kiremt*, since *Bega* is shorter in the southwest and longer in the northern and northeastern parts of the region. Variations in trends may vary with topography and also with the distance from Lake Tana [17]. Most of the rain falls in the afternoons and evenings due to the dominance of convective systems [18].

The BNB region has undergone major changes in land use due to policy changes during the 20^th^ Century, particularly in the form of deforestation [19], [20]. This is partly due to an increased population and associated increasing need for food production. As in many other African regions, the demand for agricultural land is also increasing due to foreign investors [21]. Soil and water resources need to be managed sustainably to be able to increase yields on existing agricultural lands; rather than from clearing forests and woodlands to expand output [22]. Flows in Sudan and Egypt remain a charged transboundary issue as well. With many people dependent on the flow regimes of the Blue Nile, there is an urgent socio-political need for understanding how future climate changes might influence the *Kiremt* and its associated *Bega*.

An important, but underutilized source of information on the variation in timing, intensity and duration of *Kiremt*, or the associated *Bega* in the upper BNB is the observational record that is presently over 50 years long. This oversight exists despite its importance for subsistence agriculture in the region and associated vulnerability to negative impact of rain variability. The objectives of this study were to (1) investigate the summer rains (*Kiremt*) and dry season (*Bega*) with respect to their timing, duration and intensity over an up to 50 year period (1950–2004), (2) to predict the potential impact of climate change on the summer rains and dry seasons for a future 50 year (2050–2100) scenario and, (3) to explore the use of a simple spatial model for describing rain pattern across the upper BNB.

## Materials and Methods

### The Upper Blue Nile Basin

**Figure 1:**
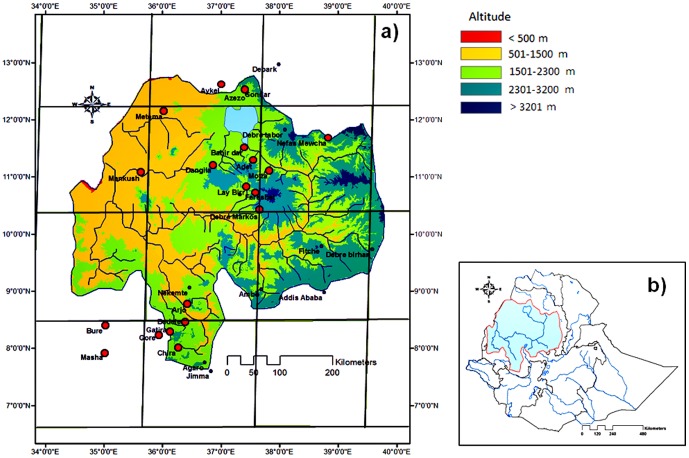
Location of (a) meteorological stations within the upper Blue Nile Basin (b) the region within Ethiopia. The altitudinal ranges are divided in accordance with the national agro-climate zonation.

The upper Blue Nile Basin (Ethiopia) stretches from 34°33′–39°45′ E and 7°49′–12°42′ N and is one of the major tributaries of the Nile River (Fig. 1). It covers *ca*. 180,000 km^2^ [23]. There are three broad topographical divisions: the highland plateaus, steep slopes adjoining the plateaus that tilt to the west and the western lowlands with gentle topography comprising the remainder of the Basin. The steep slopes and the plateaus are at 1500 m to *ca.* 4000 m a.s.l. There are three sub-basin rivers within the Nile Basin flowing from the Ethiopian highlands; the *Abbay*, *Tekeze*, and *Baro-Akobo.* In this study, Blue Nile refers to the *Abbay* flowing to Sudan and its catchment within the BNB. Even though the BNB comprises only 6.7% of the entire area of the Nile basin, the flow from the Blue Nile accounts for *ca.* 62% of the Nile water at Aswan, Egypt [23]. The three rivers from the Ethiopian highlands together account for 86% of the total flow of the Nile.

The water flow of the Blue Nile River is characterized by seasonal variability, with 82% of the annual flow occurring from July to October. The mean annual rainfall in the BNB ranges from 800 to 2200 mm [18]. The average minimum and maximum temperatures are 11°C and 26°C, respectively.

The presence of the ITCZ over the region, which is strongly linked to the El Niño-Southern Oscillation (ENSO), has proven to be relatively predictable [16]. The influence of global warming on the ENSO period’s amplitude and pattern are expected to be small [24], and therefore the influence on *Kiremt* is likely to be small.

A decline and variability of large river flow regimes have been observed during recent decades [20], [25]. There was, however, also a decrease in rainfall during the same period (mid 1960s to late 1980s). Dry years were associated with low values of the Southern Oscillation Index [26]. River flow extremes within the BNB have also been found to follow decadal patterns influenced by conditions over the Pacific Ocean [27].

### Rainfall Data

A network of 19 meteorological stations was chosen to represent the upper BNB (Fig. 1, Table 1). These were selected to cover a large range of time, space and altitude within the region. There was, however, a limitation in the distribution of rainfall stations in this study as there were only few stations in the western part of the region. The rain gauges were located between 678 and 2980 m a.s.l. and were fitted in the grid used by ECHAM5/MP1-OM, which is a coupled climate model consisting of an atmospheric General Circulation Model (GCM) and MPI-OM ocean-sea ice component. Daily total rainfall data were provided by the National Meteorological Service Agency of Ethiopia (NMSA). The time of data records for the meteorological stations were in average 24 years, ranging from 3 to 52 years and covering the period 1952–2004.

**Table 1:** Meteorological stations used in the study.

Site	Class	Longitude	Latitude	Altitude	Period for data record	Missing data
		[°]	[°]	[m a.s.l.]		[%]
Chira	1	36.16	7.44	1969	1 Jan 1985–31 Jul 2004	2.0
Masha	1	35.05	7.60	2010	1 May 1975–30 Jun 2004	11.2
Gatira	1	36.20	8.02	2110	1 Jan 2001–1 May 2004	0.0
Bedele	1	36.21	8.27	1992	14 Feb 1967–31 Dec 1968	4.4
					1 Jan 1987–30 May 2004	1.1
Gore	1	35.53	8.15	2006	1 June 1952–31 Oct 2004	2.5
Bure	1	35.08	8.27	1660	8 Feb 1975–31 May 2004	16.4
Arjo	1	36.45	8.75	1570	1 May 1972–31 Jul 2004	8.6
Debre Markos	1	37.44	10.20	2446	1 Nov 1953–30 Sep 2004	1.2
Feres Bet	4	37.35	10.51	2980	1 Feb 1980–30 Dec 2000	38.0
Lay Bir	1	37.12	10.63	2737	1 Jan 1989–30 Sep 2004	5.7
Motta	1	37.87	11.08	2400	1 Jan 1989–30 Sep 2004	3.7
Adet	1	37.29	11.16	2207	12 Sep 1986–31 Jul 2004	2.4
Bahr Dar	1	37.25	11.36	1770	5 Jan 1961–31 Oct 2004	1.1
Nefes Mewchia	1	38.45	11.73	2057	1 Oct 1957–7 Sep 1963	38.4
					1 Feb 1968–30 Nov 1970	47.3
					17 Jun 1986–31 Aug 1989	0.0
					7 Oct 1991–30 Jun 2004	3.5
Dangila	1	36.51	11.14	2102	4 Jun 1987–31 Oct 2004	4.9
Manakush	1	35.28	11.28	678	5 Jun 1984–31 May 1986	0.3
					8 Jul 1998–31 Aug 2004	6.0
Azezo	1	37.26	12.31	1960	1 Jun 1952–31 Oct 2004	7.9
Aykel	1	37.03	12.32	2238	8 Feb 1968–31 Dec 1970	60.6
					1 Jun 1980–30 Jun 2004	5.7
Metama	1	36.26	12.97	725	1 May 1971–5 Apr 1977	4.6
					1 Jun 1986–31 Dec 1989	7.1
					7 Aug 1994–31 May 2004	18.7

### Criteria for Characteristics of the Seasons

The spatial pattern of monthly rainfall was investigated using linear regression against coordinates. The onset, duration and cessation of the *Kiremt* were characterized using a previously proposed approach [15], [28]. The onset of *Kiremt* was classified as the first rain day of at least three consecutive rain days resulting in a total rain of 20 mm or more. Other criteria were that the following 30 days had no period of eight or more consecutive days without rain. The cessation day was determined as the last rain day before a dry-spell lasting at least 20 days. The temporally averaged *Kiremt* and *Bega* characteristics were correlated spatially to an index *I* defined as the Longitude divided by the Latitude. Only complete years of data record were used and no gap editing was made to produce the averages on the season characteristics.

The onset of the *Belg* was determined in a similar way as the *Kiremt* but as the first three consecutive rain days resulting in a total rain of 5 mm or more. The *Bega* season was thereafter classified as the number of rain-free days between the cessation of *Kiremt* and onset of *Belg*.

### Rain Frequency

Spatial and temporal variation of observed rainfall were analyzed for changes in rain frequency and intensity for complete years in two sites (Debre Markos and Azezo) between 1954–1977 and 1978–2001 with plots of Probability of Excedence (PE) for rain days of a certain range of magnitude. The sites were chosen to represent differences found within the region and to have a long and consistent time series of rain data. These periods were chosen to be of the same length and to correspond with the wet and dry anomalies of the *Kiremt* rain within the BNB [29] with the earlier period being wet and the latter dry. The PE was calculated according to:
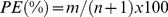
(1)


Where *m* is the rank order of each rain day (*m* = 1 as the largest event and *m* = *n* as the lowest event) and *n* is the total number of days [30].

### Climate Scenario and Downscaling

Rainfall data from the ECHAM5/MP1-OM scenario was used in this study. The data were obtained from the Climate and Environmental Retrieving Archiving (CERA) database provided by the World Data Center for Climate, Hamburg (WDCC), (http://www.mad.zmaw.de/wdc-for-climate/). The model output was prepared for the Intergovernmental Panel on Climate Change (IPCC) Fourth Assessment climate of the 20th Century experiment (20C3M) and the scenario contains only anthropogenic forcing. Data from 12 grid-boxes (each 2°×2°) was extracted to cover the upper BNB. Seven of these grid-boxes covered areas from which observed rain data were available.

The daily rain from observations made between 1952 and 2004 was used to spatially and temporarily down-scale the ECHAM5/MP1-OM scenario in order to produce daily rain data for the period 2050–2100 for each of the 19 stations. As a first step the observed monthly rain data from all stations were correlated to the reference data of the corresponding grid-box from ECHAM5/MP1-OM (1952–2004). Next, the correlation between observed and ECHAM5/MP1-OM data was thereafter calibrated by stepwise changes in the trend coefficient and the intercept. The calibrated correlation was projected to the future climate data assuming that the daily variability in the ECHAM5/MP1-OM data in the future projection will be the same as for the observed period. The daily variation was implemented using a delta change method for precipitation [31]–[33]. The daily variation used was that of the reference period (1952–2004). A rain day scaling factor, *f_prec_*, was derived for each month, by dividing the average differences in monthly total rainfall between the ECHAM5/MP1-OM predicted data *P_pred_* and observed data *P_obs_* by the average number of rain days *n_dprec_* for each month:
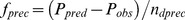
(2)


The scaling factor was added to the reference data for each rain day. The downscaled model data were used to investigate potential future changes in correlations to latitude and longitude as well as arrival, duration, departure and intensity of *Kiremt* and the number of rain-free *Bega* days.

## Results

**Figure 2:**
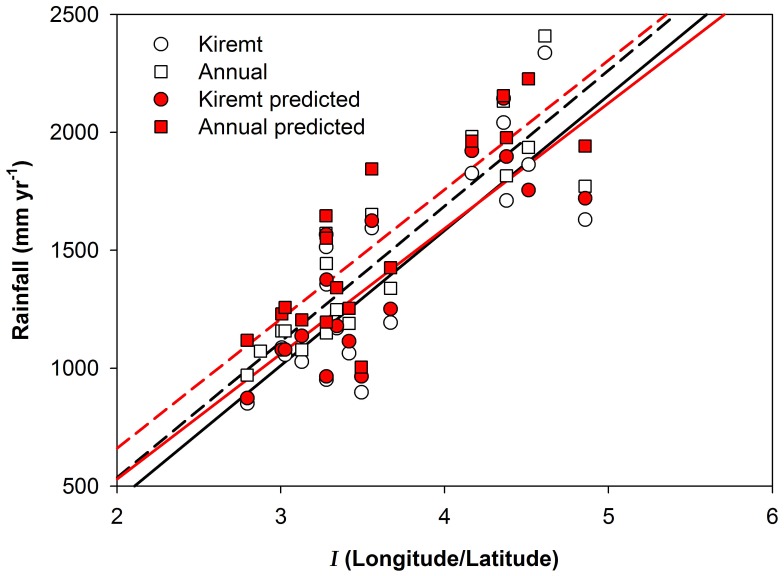
Linear correlation of *Kiremt* (circles) and annual rainfall (squares) to the index of coordinates, *I (Longitude/Latitude)*. Open symbols represent observed rainfall 1952–2004 and filled symbols represent predicted rainfall for 2052–2100.

The annual mean rainfall for the selected stations and time ranges over the area (3–52 yrs, 35°05′–38°45′ E and 7°44′–12°97′ N) was 1470 mm, spatially ranging between 970 mm in the northwest (Metema) to 2410 mm in the southwest (Masha), of which the *Kiremt* rainfall represented on average 93% (Table 2). Average annual rainfall correlated (R^2^ = 0.75) to the index of map coordinates (*I)*. The proportion of *Kiremt* rainfall was relatively consistent (Fig. 2 and Tab. 2) despite the spatial difference in average annual rainfall over the region. Both spatial and temporal variations of rainfall were found in the time series of daily rainfall.

**Table 2:** Observed rainfall data (1952–2004) and predicted rainfall data (2052–2100).

Site	Index [Long/Lat]	Yearly precipitation [mm]		Kiremt precipitation [mm]		No. Kiremt days [days]		Kiremt rain intensity [mm day^−1^]		No. Bega days [days]	
		obs.	pred.	obs.	pred.	obs.	pred.	obs.	pred.	obs.	pred.
Chira	4.86	1770	1940	1630	1720	322	287	6.0	6.0	60	47
Masha	4.61	2410	2430	2190	2310	269	274	8.3	8.5	42	44
Gatira	4.51	1940	2230	1860	1760	271	250	6.9	7.1	80	87
Bedele	4.38	1820	1980	1710	1900	215	233	8.0	8.3	103	96
Gore	4.36	2130	2160	2040	2140	276	271	7.5	8.0	71	83
Bure	4.24	1350	1480	1250	1350	226	229	5.6	5.9	113	112
Arjo	4.17	1980	1960	1830	1920	209	224	9.2	9.0	110	108
Debre Markos	3.67	1340	1430	1190	1250	181	187	6.8	6.9	103	126
Feres Bet	3.56	1650	1840	1590	1620	212	215	7.4	7.4	115	126
Lay Bir	3.49	980	1000	900	960	197	161	4.6	6.1	136	129
Motta	3.42	1190	1250	1060	1110	175	193	6.4	6.1	143	136
Adet	3.34	1250	1340	1170	1180	196	196	6.1	6.2	147	140
Bahr Dar	3.28	1440	1550	1360	1380	159	163	8.7	8.6	182	181
Nefes Mewchia	3.28	1150	1200	950	960	154	161	6.3	6.1	163	158
Dangila	3.28	1570	1650	1510	1570	196	213	7.9	7.4	149	135
Manakush	3.13	1080	1200	1030	1140	156	149	6.6	7.6	209	204
Azezo	3.03	1160	1260	1060	1080	169	169	6.4	6.5	158	157
Aykel	3.01	1160	1230	1090	1080	173	169	6.4	6.4	175	175
Metama	2.80	970	1120	850	870	136	135	6.3	6.6	208	216
**Average**	**3.71**	**1490**	**1590**	**1380**	**1440**	**205**	**204**	**6.9**	**7.1**	**130**	**129**

### Temporal Patterns of Rainfall

**Figure 3:**
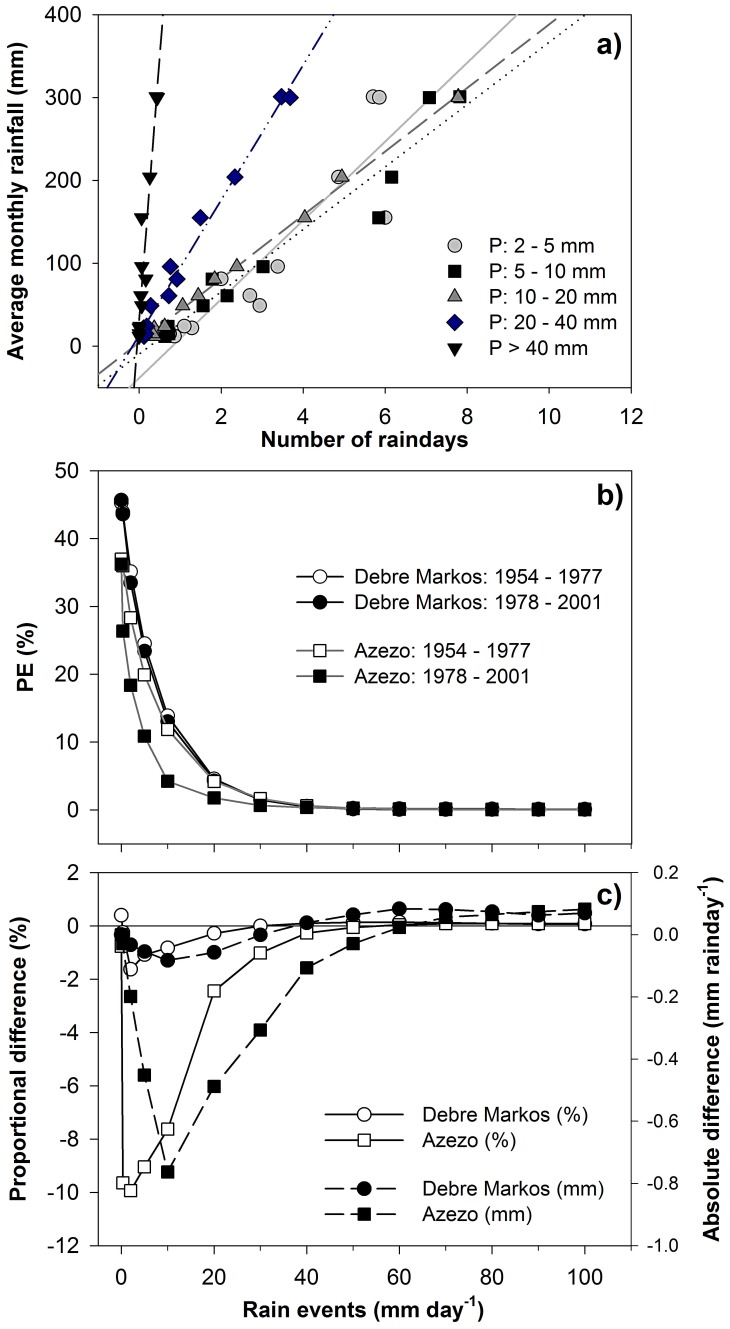
Rain distribution (a) and rain frequencies (b and c) in Debre Markos and Azezo. Rain day distribution of certain magnitudes (5–10, 10–20, 20–40 and >40 mm) were used for the period 1954–2004 in Debre Markos. For the Probability of Exceedence (PE) and proportional (%) and absolute (mm/rain day) difference in PE (in Debre Markos and Azezo), a “dry” period (1954–1977) and a “wet” period (1978–2001) was used.

The temporal patterns were correlated to frequency distributions; the higher the monthly total rainfall, the higher the number of rainy days. For example, in Debre Markos the correlation between the number of rain days between 2 mm and 5 mm and the monthly total rain was 82%. This linear correlation varied for different rainfall ranges and also increased with the higher ranges (Fig. 3a). The annual rainfall within the region slightly decreased over the second half of the 20^th^ century, though this decrease was not found everywhere in the Basin. For example, the differences in PE between the “wet period” 1954–1977 and the “dry period” 1978–2001 were larger in the more northerly Azezo than in Debre Markos (Fig. 3b–c). In Azezo, there was a change to drier days, whereas in Debre Markos these became fewer. In both sites the number of days having 5–30 mm decreased. That change was more pronounced in Azezo. There were no changes in the more extreme events (≥50 mm day^−1^). There was a difference in PE over the region, with a gradual decrease of rain days ≥40 mm day^−1^ (data not shown) towards the northeast. This gradual decrease coincided with the timing and duration of *Kiremt* as determined by the ITCZ movement over the region.

### Spatial Patterns of Rainfall

**Figure 4:**
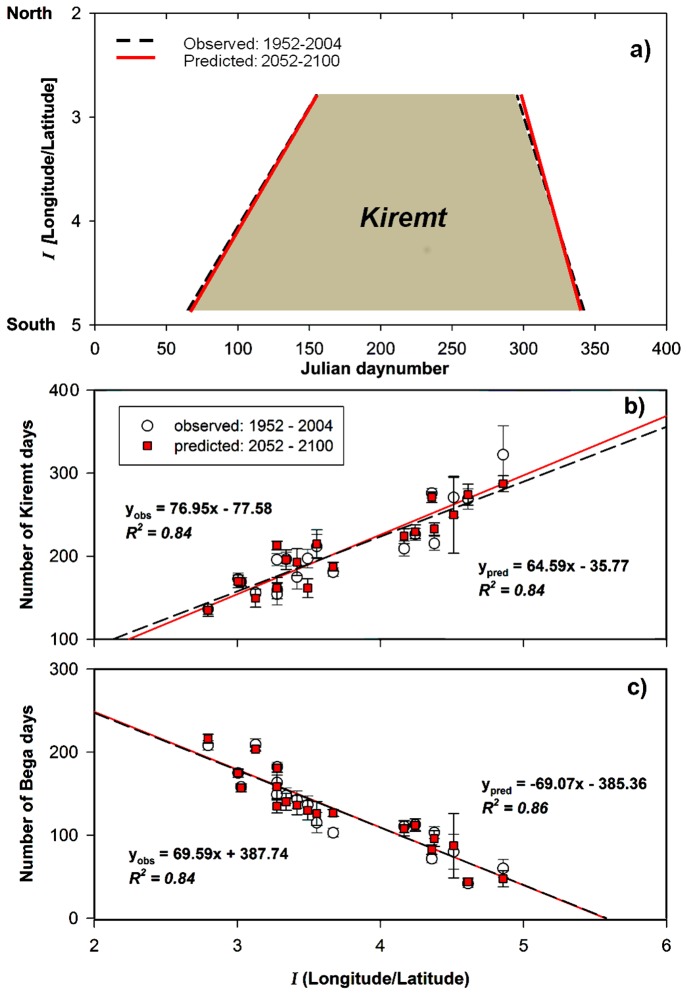
Presence of *Kiremt* and duration of *Kiremt* and *Bega* correlated to the index of coordinates, *I*. The correlation represents observed data (1952–2004) in circles and predicted data a century later (2052–2100) in squares over the upper Blue Nile Basin.

The correlation of rainfall to *I*, the ratio between longitude to latitude, was also seen in the average monthly total rainfall except for July to September, *i.e.* during the *Kiremt* (data not shown). Indeed, there was a clear southwest-northeast component in the onset, duration and cessation of *Kiremt* that could be explained by *I* of the rain gauge, expressed with simple linear functions (Fig. 4a–c). There are, however, limited number of rainfall stations in the western and eastern parts of the BNB, whereas the available data represents clusters of rainfall stations from the southwest to the northeast. The onset of *Kiremt* was negatively correlated to *I* (88%), a lower value being more to the north-northeast and a higher value to the south-southwest) and occurred between 6 March–7 June. The number of *Kiremt* days and the cessation of the *Kiremt* were also related to *I* (83 and 51% respectively). The mean length of the *Kiremt* season varied between 136 and 322 days over the region (Table 2) with a longer *Kiremt* towards the southwest.

**Figure 5:**
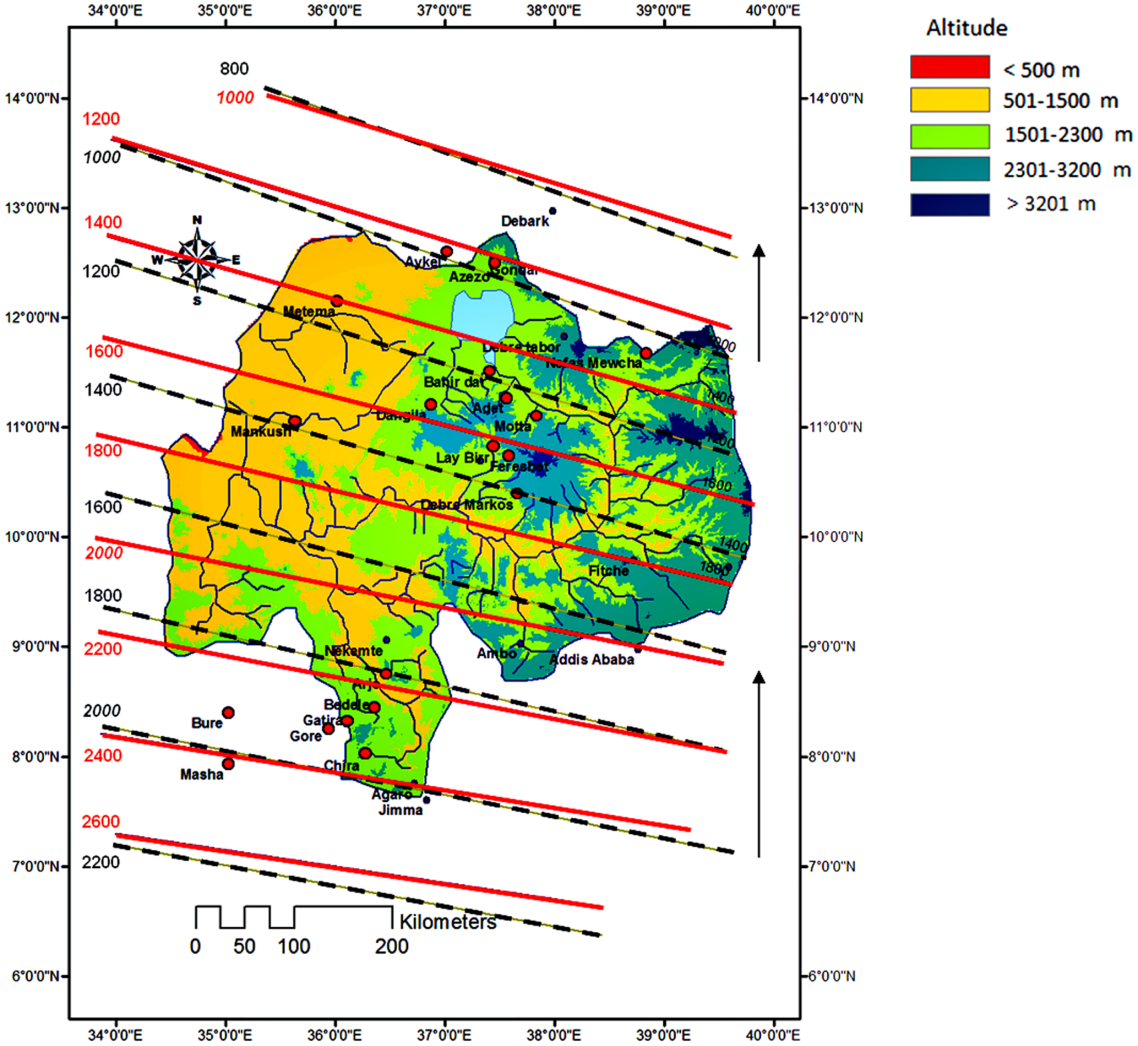
Estimated isohyets for 1952–2004 (dashed line) and 2052–2100 (solid line) annual rainfall. Arrows indicate the predicted northward shift in isohyeths.

The spatiotemporal mean *Kiremt* started on the 25^th^ of April and lasted for 205 days (12^th^ November) with a rain intensity of 6.9 mm/day, ranging from 4.9 to 8.8 mm/day (Table 2). The spatiotemporal mean number of rain-free *Bega* days was 130 days. The *Bega’s* length ranged from 42 days in the southwest to 209 days in the northwest (Table 2). The largest number of rain-free *Bega* days was in the northwesterly sites Manakush and Metama. In a similar way as for *Kiremt,* the number of rain-free *Bega* days correlated linearly (R^2^ = 84%) with *I* (Fig. 4c). A simple spatial model for the presence of *Kiremt* is presented in Fig. 4a and in addition one for annual total rainfall over the region could be developed by using the relationships found between rainfall and *I* (Fig. 5).

### Downscaling of Monthly Rainfall

The correlation of rainfall from each individual site to rainfall from the corresponding ECHAM grid box was in all cases calibrated by stepwise change of the trend coefficient and the intercept. The improvement in trend coefficient and intercept also increased the correlation coefficient in all sites, from a range of 37–76% to a range of 57–86% (Table 3).

**Table 3:** Linear regression model of monthly rainfall based on observed data and model (n = number of months, a_1_ = trend, a_0_ = intercept (mm), and R^2^ = determination coefficient).

Site	n	a_1_	a_0_	R^2^
Chira	231	0.99	1.2	0.57
Masha	286	0.98	4.5	0.59
Gatira	39	0.99	0.3	0.82
Bedele	199	0.97	8.1	0.69
Gore	607	0.99	2.5	0.72
Bure	287	0.95	8.9	0.65
Arjo	336	0.97	10.1	0.67
Debre Markos	594	0.99	2.2	0.73
Feres Bet	153	0.99	2.1	0.82
Lay Bir	181	0.97	6.1	0.71
Motta	186	0.98	5.5	0.74
Adet	211	0.97	8.1	0.72
Bahr Dar	523	0.95	17.7	0.60
Nefes Mewchia	249	0.98	5.5	0.70
Dangila	202	0.98	5.7	0.81
Manakush	92	0.97	6.8	0.86
Azezo	576	0.97	7.8	0.72
Aykel	293	0.97	7.8	0.77
Metama	169	0.95	12.5	0.71

**Figure 6:**
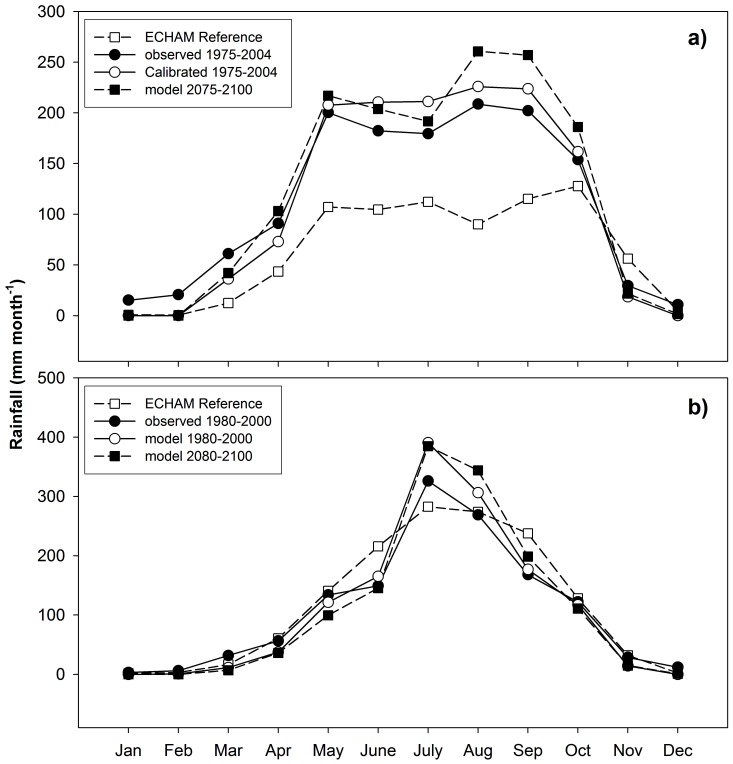
Example of variation of monthly rainfall for two stations (a) Bure and (b) Adet. Observed data (filled circles), reference data from corresponding ECHAM5 grid box for same period (open squares), calibrated model for corresponding ECHAM5 grid box and period (open circles), as well as predicted rainfall for a century later (filled squares).

Aspects of the downscaled variation in the mean monthly observed rainfall over the year are presented for two stations, Bure in the southwest and Adet in the northeast part of the Basin (Fig. 6) i) the observed rainfall, ii) the monthly control data for the corresponding ECHAM5/MP1-OM grid box, iii) the calibrated model of past rainfall (spatially downscaled from the ECHAM5/MP1-OM data and iv) the downscaled predicted rainfall. The upper panel shows the typical bimodal annual trend in rainfall for the southwestern part of the region, with one peak of rainfall in May and another in August – September. Both peaks were associated with the presence of the ITCZ. For the same reason, there was only one peak of rainfall (July – August) in the more northeasterly part of the region (lower panel). The control model of the ECHAM5/MP1-OM data captured the trends over the years in both types of annual character for the control period (1950–2000), but underestimated the rainfall over the dry *Bega* months and overestimated the *Kiremt* rain, especially in Bure.

### Predicted Rainfall

The spatiotemporal annual mean rainfall (3–52 yrs) over the region (35°05′–38°45′ E and 7°44′–12°97′ N) was predicted to be 1590 mm by the second half of this century, which is an increase of 100 mm (6.4%). Even if the spatiotemporal annual mean of *Kiremt* rain is predicted to increase by 3.8%, the regional proportion of *Kiremt* is predicted to be 89.5% of annual rainfall, which is a decrease by 3.2% (Fig. 2 and Table 2). However, the proportion of *Kiremt* rainfall increased by 2.0–5.5% in five sites (Masha, Bedele, Gore, Arjo and Lay Bir), with most of these in the south of the region. The predicted *Kiremt* rainfall ranged between 870 to 2310 mm, which corresponds to a change ranging from −110 to 190 mm/year. There was a linear correlation between *I* and the predicted change in *Kiremt* rainfall relative to the present (R^2^ = 51%, excluding data from Gatira which was an outlier in this relation). The change in *Kiremt* rainfall is expected to increase towards the southwest and only slightly increase (or decrease) towards the north (data not shown).

The onset of *Kiremt* is expected to remain similar to present conditions (Fig. 4b), and the southwest-northeast component in its progress is also predicted to remain as it has been in the latter half of the 20^th^ Century. A slight decrease in the number of *Kiremt* days was found towards the northeast, while an increase was found towards the southwest due to the delayed cessation of *Kiremt* (Fig. 4a and b). The largest increase in the number of *Kiremt* days, at an individual site, was 18 days and the largest decrease was 36 days (Fig. 4b). Rainfall intensity was inversely correlated to the *Kiremt* length; rainfall intensity decreased with the longer *Kiremt* (−0.3 mm/day) and increased with the shorter *Kiremt* (+1.4 mm/day). The spatiotemporal mean *Kiremt* is predicted to start on the 24^th^ of April and last for 204 days (13^th^ of November) in 2050–2100. The spatiotemporal mean number of rain-free *Bega* days is predicted to remain similar to present conditions. The spatial range in the number of rain-free *Bega* days was, however, predicted to increase by 7 to 44 days in the southeast and up to 216 days in the north (Table 2). The correlation in the number of rain-free *Bega* days with *I* (R^2^ = 86%) is similar to past conditions (Fig. 3d). The predicted increase in the annual rainfall over the region resulted in a *ca*. 1.1° (120 km) northward shift of the northerly 1000 mm annual rain isohyet and a *ca*. 1.7° (190 km) northward shift of the southerly 2000 mm isohyet (Fig. 5).

## Discussion

The spatiotemporal trends predicted for the future rainfall were consistent with those found in the past half-century (Figs. 2 and 4). With this in mind it is possible to conceptualize the past and potential future variability in rainfall and adapt water management and land use to future climate across the upper BNB.

Earlier reports on the proportions of annual rainfall comprised by *Kiremt* vary from 70 to exceeding 75% [16], [18], [26]. In this study the spatiotemporal average *Kiremt* (within 35° 05′–38° 45′ E and 7° 44′–12° 97′ N during 1952–2004) was 93% of the annual rainfall (Fig. 2 and Table 2). The criteria for determining the *Kiremt* duration will highly influence that proportion. However, as rainfall varies spatially, the location and size of the study area may also influence the result. The large proportion of annual rainfall during *Kiremt* is reflected by the 82% of the annual flow in the Blue Nile occurring during *Kiremt* season. The onset of *Kiremt* was more pronounced than cessation which was more difficult to identify in the southern and southwesterly stations where there was a more diffuse transition of *Belg* into *Kiremt*. It is apparent that spatiotemporal scales and methodologies need to be taken into account when comparing studies.

The total rainfall decreased during the last quarter of 20^th^ century (or 1978–2001) compared to the preceding quarter as reported elsewhere [29]. This decrease did not appear to be homogenous for the area as there was a larger decrease in the north (Fig. 3b–c). It is, however, not necessarily the total rainfall that is most important for water resource management. The timing of onset and cessation of rains may be more important for rain-fed agriculture in order to match the growing period of the staple crops. The spatial pattern in monthly frequency distribution was exploited to improve the prediction of future rainfall (Fig. 3a–c). The consistent correlation of the number of rain days of a certain magnitude (exemplified in Fig. 3a) could be useful for temporal downscaling of monthly rainfall data together with a statistical method for distributing rain days over each month.

The number of rain-free *Bega* days has major impacts on crop production. During the last 50 years the regional difference was large in the number of rain-free *Bega* days. In the north, there were 167 more rain-free days during *Bega* than in the south (Fig. 4c and Table 2). It has previously been found that the number of *Bega* rain days have been rather stable during the last 30 years in the Ethiopian highlands (using other rain stations than in this study) [28].

Comparing predicted weather for present conditions with observed weather is a way to verify GCM-scale scenarios. Uncertainties of model-based predictions of future climate and its impacts are, however, by nature unavoidable and are significant in the upper BNB [2]. It has been suggested that, despite existing uncertainties, climate change scenarios are suitable to explore the sensitivity of a range of African environmental systems [34]. The uncertainties of climate scenarios need to be communicated to policy-makers, in order that they are aware of the uncertainties of the information on which their policy is based [2], [35].

A number of global climate models are available to study the effects of climate change on the hydrological regime of the upper BNB. Results from seven GCMs for Ethiopia indicated variability in predicted 2050 rainfall from a decrease of 10% to an increase of 25%, as well as an increase in annual air temperature by 0.8 to 4.3**°**C compared to 1961–1999 long-term means [34]. In other studies a mild increase in rainfall over the BNB is expected in the 2050s with a spatial mean increase over all seasons of *ca.* 11% [36], [37]. The ECHAM5/MP1-OM scenario was found to give the best result [38] from among 23 GCMs (both in terms of rainfall patterns, timing and magnitude) when comparing 50 years of monthly mean rain data over the upper BNB with the 0.5° observation data from the Climate Research Unit (CRU TS2.1; [39]). This is why we based the modeling of the future in this on the ECHAM5/MP1-OM scenario.

The small increase in annual rainfall predicted over the coming 50 years did not appear to be evenly distributed over the region. There was a larger northward shift in the southerly isohyets than in the northerly ones (Fig. 5). The mild increase in rainfall over the region in 2050–2100 is in agreement with other findings [37]. The length of *Kiremt* was expected to increase in the south and slightly decrease in the north, however, the regional total *Kiremt* rain was balanced by a decrease in rain intensity with the longer *Kiremt* in the south and an increase in intensities associated with the shorter *Kiremt* in the north (Fig. 4a and b). The spatiotemporal average of rain-free *Bega* days is expected to remain similar in the future except for a slight increase in the spatial difference which might influence the cultivation zones of different crops.

The perceived correlation between indices of map coordinates (*I*) and characteristics of *Kiremt* rainfall can be valuable for water resources planning and management in a region where data availability is limited. It is, however, necessary to consider that the established patterns are built on limited data. The available meteorological stations resulted in a clustered distribution over the area, had different periods of data record and may have had variations in the quality of data. Nevertheless, perceived distinctive spatial zonation was found in the correlation of *Kiremt* rainfall characteristics and the number of rain-free *Bega* days to *I* (Fig. 4c). This spatial zonation in climate and climate change across the BNB needs to be communicated rather than just allowing planning to be based on regional averages that ignore clear patterns across the region. This knowledge may improve and encourage different adaption strategies for farmers within the BNB [20], [40]. Factors influencing farmers’ decisions to adapt to climate change in Ethiopia were found not only to be wealth, access to extension and credit, but also information on climate [41].

We present a simple spatial model for communicating the length of the rainy period and dry season, as well as the amount of rain falling during the rainy season (Fig. 4, 5 and 6). This could be useful for the long-term planning of water resource management within the region. Even if the presented model of annual rainfall distribution over the upper BNB does not take the topography over the region into account, the results match previously mapped distribution [16] in terms of magnitudes and the southwest-northeast zonation. Considering the consistent linear correlation of coordinates and rainfall it could be possible to validate and refine the presented model by including data from a network of short-term and high temporal resolution rain gauges.

The large weather systems generating the seasonality in rainfall over the region have been relatively stable and this is expected to stay the same with global warming as it is currently understood. This is important for livelihoods in the region, since the degree of inter-annual variability that already exists is rather large [6] and very costly for the welfare of the region [42], [43]. It has been concluded that the inter-annual variability in the timing and amount of the rains is of a magnitude that significantly impairs the productivity of the region [3]. Future studies should try to determine in what ways increased population pressures and associated land use/land cover changes may affect the sensitivity to climate variability. Information from the present study could be used in analyzing the possible variation in sensitivity to rainfall between the different characteristic zones of rainfall over the upper BNB.

### Conclusions

Over the latter half of the 20^th^ Century the amount of *Kiremt* rains and the length of the *Bega* dry season have exhibited consistent spatial patterns over the BNB. We demonstrated that there is a distinct regional trend from the southwest to northeast in the amount and duration of the *Kiremt* rains, as well as the timing of the transition into the *Bega* dry season. These patterns could be explained by simple spatial models. These patterns were predicted to be maintained in the future but with slight changes that also vary across the region. The results highlight that when planning water management and climate adaptation within the upper BNB there is a need to account for the characteristic zonation of rainfall across the region, and not just the total for the whole region, especially when conceptualizing climate adaption and potential consequences of a changed climate.

We recommend that simple spatially resolved models based on historical trends, like the one presented here, can be used as a baseline for the long-term planning of the water resource management. However, the inter-annual to inter-decadal variability will remain, with all its potential consequences, for rain-fed agriculture.
